# Preliminary Effectiveness of a Telehealth-Delivered Exercise Program in Older Adults Living With and Beyond Cancer: Retrospective Study

**DOI:** 10.2196/56718

**Published:** 2025-01-13

**Authors:** Emily R Dunston, Sonal Oza, Yang Bai, Maria Newton, Leslie Podlog, Kish Larson, Darren Walker, Rebecca W Zingg, Pamela A Hansen, Adriana M Coletta

**Affiliations:** 1 Department of Health and Kinesiology, University of Utah, Salt Lake City, UT, United States; 2 Department of Rehabilitation Medicine, Emory University School of Medicine, Atlanta, GA, United States; 3 Faculty of Medicine, School of Kinesiology and Physical Activity Sciences, Université de Montréal, Montréal, QC, Canada; 4 CHU Sainte-Justine Research Center, Montréal, QC, Canada; 5 Huntsman Cancer Institute, Salt Lake City, UT, United States; 6 Division of Physical Medicine and Rehabilitation, University of Utah, Salt Lake City, UT, United States; 7 Cancer Control and Population Sciences Program, Huntsman Cancer Institute, University of Utah, 2000 Circle of Hope Drive, Research South Building Rm 4747, Salt Lake City, UT, 84112, United States, 1 801-213-6012

**Keywords:** physical activity, physical function, telerehabilitation, remote exercise, digital health, cancer survivors, older adults, smartphone

## Abstract

**Background:**

Exercise can attenuate the deleterious combined effects of cancer treatment and aging among older adults with cancer, yet exercise participation is low. Telehealth exercise may improve exercise engagement by decreasing time and transportation barriers; however, the utility of telehealth exercise among older adults with cancer is not well established.

**Objective:**

We aimed to evaluate the preliminary effectiveness of a one-on-one, supervised telehealth exercise program on physical function, muscular endurance, balance, and flexibility among older adults with cancer.

**Methods:**

In this retrospective study, we analyzed electronic health record data collected from the Personal Optimism With Exercise Recovery clinical exercise program delivered via telehealth among older adults with cancer (≥65 y) who completed a virtual initial program telehealth assessment between March 2020 and December 2021. The virtual initial assessment included the following measures: 30-second chair stand test, 30-second maximum push-up test, 2-minute standing march, single leg stance, plank, chair sit and reach, shoulder range of motion, and the clock test. All baseline measures were repeated after 12-weeks of telehealth exercise. Change scores were calculated for all assessments and compared to minimal clinically important difference (MCID) values for assessments with published MCIDs. Paired samples *t* tests (2-tailed) were conducted to determine change in assessment outcomes.

**Results:**

Older adults with cancer who chose to participate in the telehealth exercise program (N=68) were 71.8 (SD 5.3) years of age on average (range 65‐92 y). The 3 most common cancer types in this sample were breast (n=13), prostate (n=13), and multiple myeloma (n=8). All cancer stages were represented in this sample with stage II (n=16, 23.5%) and III (n=18, 26.5%) being the most common. A follow-up telehealth assessment was completed by 29.4% (n=20) of older adults with cancer. Among those who completed a follow-up telehealth assessment, there were significant increases in the 30-second chair stand (n=19; mean change +2.00 repetitions, 95% CI 0.12 to 3.88) and 30-second maximum push-up scores (n=20; mean change +2.85 repetitions, 95% CI 1.60 to 4.11). There were no significant differences for the 2-minute standing march, plank, single leg stance, sit and reach, shoulder mobility, or clock test (*P*>.05). Nine (47.3%) older adults with cancer had a change in 30-second chair stand scores greater than the MCID of 2 repetitions.

**Conclusions:**

Our findings suggest a one-on-one, supervised telehealth exercise program may positively influence measures of physical function, muscular endurance, balance, and flexibility among older adults with cancer, but more adequately powered trials are needed to confirm these findings.

## Introduction

Adults aged 65 years and older currently account for 67% of cancer survivors (eg, individuals living with and beyond a cancer diagnosis) in the United States [1]. By 2040 it is projected that 73% of cancer survivors in the United States will be aged 65 years and older [2]. Cancer treatment compounds the normal effects of aging resulting in an accelerated aging effect [3]. A hallmark characteristic of accelerated aging is poor physical functioning [3]. Older adults with cancer experience worse physical function than their younger counterparts [4] and older adults without cancer [5,6]. Physical function plays a critical role in the health of older adults with cancer and poor function is associated with decreased cancer survival [7], increased all-cause mortality [8], and increased symptom severity [9].

Regular participation in exercise is one strategy to help mitigate declines in physical function among cancer survivors of all ages. Among older adults with cancer, individualized, in-person supervised exercise programming, including combined aerobic and resistance training, for at least 12-weeks significantly improves physical function [10,11], quality of life [12], muscular strength [10,11], aerobic endurance [10,11], and symptoms of anxiety and depression [11]. Despite the numerous benefits of exercise for older adults with cancer, participation in exercise in this population is low with only 12% of older cancer survivors meeting both the aerobic and strength training guidelines [13]. Reasons for low engagement among this population include: lack of available exercise programming in convenient locations [14], transportation concerns [14,15], lack of time [14,16], physical symptoms (eg, fatigue) [14,16,17], and comorbidities [14,17]. Strategies to reduce barriers to participating in exercise among older adults with cancer are needed to improve exercise engagement and physical function in this population.

Delivering exercise programs using telehealth is a useful strategy in attenuating these barriers. Telehealth delivery of exercise detaches the exercise program from a physical location, resulting in exercise engagement in a more convenient location, such as the home, eliminating the need for travel, and reducing overall time commitment [18]. Telehealth delivered exercise can also lower the cost of participation as participants do not need to pay for transportation or parking [18]. After transitioning two trials from in-person to telehealth exercise, due to the impact of the COVID-19 pandemic on in-person research, Winters-Stone et al [19] observed better adherence and retention for telehealth exercise compared to in-person exercise among adult cancer survivors of all ages. In addition to addressing these barriers, supervised, telehealth-delivered exercise programs among adult cancer survivors of all ages have demonstrated improvements in physical symptoms and comorbidities such as: physical function [20,21], aerobic endurance [20,22], muscular endurance [20,22], and fatigue [22]. Specific to older adults living with cancer, telehealth delivery of exercise programming is considered acceptable [23], feasible [24], and safe [25]. Moreover, older cancer survivors view telehealth delivery of exercise positively and report limited technology related barriers to telehealth exercise participation [26]. Barriers and facilitators to participating in telehealth exercise reported by older cancer survivors are similar to those reported by their younger counterparts [26]. To our knowledge, only two studies to date have evaluated effectiveness of supervised telehealth exercise programming (ie, delivered via telehealth in real time) exclusively in older adults with cancer [24,27]. Both trials delivered group resistance training programs and observed significant improvements in markers of physical function after participating in the program [24,27]. However, little is known regarding the effectiveness of one-on-one telehealth supervised exercise in older adults with cancer. Given the dearth of research, we sought to address the issue in this investigation.

The purpose of this investigation was to explore the preliminary effectiveness of a one-on-one, supervised telehealth clinical exercise oncology program among older adults with cancer on physical function, muscular endurance, balance, and flexibility. We hypothesized that participation in telehealth exercise would result in a statistically significant improvement in physical function, muscular endurance, aerobic endurance, balance, and flexibility among older adults with cancer.

## Methods

### Study Design and Sample

This was a retrospective analysis of electronic health record data collected between March 2020 and December 2021 from the Huntsman Cancer Institute (HCI) at the University of Utah’s clinical exercise oncology program, the Personal Optimism With Exercise Recovery (POWER) program. This study was approved by the University of Utah Institutional Review Board (IRB_00072431). To be included in this analysis participants must have met the following inclusion criteria: (1) ≥65 years of age, (2) diagnosis of invasive cancer, and (3) completion of an initial POWER program assessment via telehealth. Demographic and clinical data including age, sex, race, ethnicity, cancer site, cancer stage, and cancer treatment history, were pulled from the medical record. Initial and follow-up assessment data were abstracted from the POWER program clinical database by a trained researcher (ERD) with support from certified exercise physiologists within our hospital-based exercise oncology program using a study specific spreadsheet developed in partnership with this study’s team. Data were cleaned to ensure all measures were within a physiologically reasonable range and units were consistent within measures (eg, all plank assessments were reported in seconds). Cancer treatment history from manual data abstraction was verified with the electronic health record.

### Exercise Program

The POWER program is a hospital-based exercise oncology program embedded into clinical practice at the HCI. Details of this clinical program have been previously published [28]; therefore, only pertinent details will be discussed here. While the program has traditionally been offered both in-person and via telehealth, the POWER program shifted to exclusive telehealth delivery due to the COVID-19 pandemic in March 2020 and continued to operate primarily via telehealth through December 2021. Anyone seeking care at the HCI is eligible to participate in the POWER program and patients can enroll in the program through self-referral or physician referral.

POWER provides personalized exercise prescriptions, including both aerobic and resistance training, to program participants based on an initial assessment conducted by a physiatrist and certified exercise physiologist with expertise in cancer via telehealth. The typical length of the program was 12-weeks, but varied based on participant preference. After about 12-weeks, participants were encouraged to complete a telehealth follow-up assessment to evaluate their progress (ie, reassess all baseline measures) and revise the exercise prescription to promote continued progress. Ultimately, the POWER program aims to help survivors become comfortable and capable of safely engaging in exercise independently.

The exercise prescription was individualized to each participant’s needs and was informed by the initial telehealth assessment which included a review of medical and cancer treatment history, physical examination, review of current exercise behavior, and assessment of physical function, muscular endurance, and flexibility. Following the initial assessment each participant met with a certified exercise physiologist twice weekly, via telehealth, for the duration of their program, for a supervised, 60-minute resistance training session. Body weight training and resistance bands were the primary mode of resistance training delivery; however, the resistance training program may have also included resistance machines or free weights per participant access and preference. No equipment was provided to participants by the exercise program. Prescribed aerobic exercise was completed unsupervised by each participant. The goal of each participant’s program was to work toward meeting the physical activity guidelines for cancer survivors [29].

Participants accessed the telehealth exercise visits directly through their online patient portal using any electronic device that was capable of video calls (eg, smartphone, tablet, laptop, etc). Telehealth visits were conducted directly through electronic health records (Epic Systems Corporation) which allow certified exercise physiologists easy access to the participants address, contact information, and emergency contacts. Participants’ location for each telehealth exercise session and contact information, in case the telehealth session was disconnected, was verified by the certified exercise physiologist at the start of each session. While survivors had an out-of-pocket cost of approximately US $8 per telehealth exercise training visit, the baseline and follow-up assessments were covered by medical insurance reimbursement.

### Measures

#### Overview

The following measures were included in the telehealth initial and follow-up assessments in the POWER program. When developing the telehealth assessment procedures, decisions about which measures to include were based on the feasibility of carrying out measures in a telehealth format and alignment with the in-person POWER program assessment [28]. When administering the telehealth assessments, the video camera angle was adjusted for each assessment so that the certified exercise physiologist could observe the full range of motion and ensure proper form was being used.

#### 30-Second Chair Stand Test

Lower extremity function was evaluated with the 30-second chair stand test. Participants stood from a seated position, with arms crossed across their chest, and were instructed to stand up and sit down as many times as they could in 30-seconds [30]. The number of repetitions (ie, return to seated) completed in 30-seconds were recorded. Repetitions were counted using consistent methods across assessments and assessors to optimize the reliability of this assessment. The 30-second chair stand test has been shown to be a good predictor of lower extremity function in older adults [31] and safe to conduct using telehealth in adults with cancer [32]. Moreover, the 30-second chair stand test has good test-retest reliability in older adults with cancer (intraclass correlation [ICC]=0.89) [33,34]. A minimal clinically important difference (MCID) of 2.0 has been established for the 30-second chair stand test [35].

#### 30-Second Maximum Push-Up Test

Muscular endurance was assessed using the 30-second push-up test. The starting position for push-ups was with the hands on the floor approximately shoulder width apart and arms straight. Participants were instructed to lower themselves down toward the floor until their chest was one fist width above the floor and then return to the starting position; this is one repetition. Participants were asked to complete as many push-ups as possible in 30-seconds. If the participant was unable to perform a standard push-up (on toes), they were able to modify by starting on their knees or performing wall push-ups depending on ability [36]. Any modifications made at baseline were replicated at follow-up.

#### 2-Minute Standing March

Aerobic endurance was assessed using the 2-minute standing march test. Participants stepped in place with a step height no lower than the midpoint between the patella and iliac crest. The number of steps (right and left equals one) completed in 2-minutes were recorded. If necessary, participants could use one hand on a counter-top or a chair to assist with balance. The 2-minute standing march has been shown to be a good alternative to the 6-minute walk test [37,38] with strong test-retest reliability (ICC=0.99) when assessed among older adults via telehealth [39].

#### Single Leg Stance

Balance was assessed using a single leg stance. Participants were instructed to lift one foot off of the ground and balance on one leg without holding onto anything for support for as long as possible with their eyes open. The single leg stance was performed once on each leg. Time balancing without assistance (from hands or the other foot) was recorded for each leg. No maximum time cap was imposed for the single leg stance. The single leg stance test has demonstrated good reliability (ICC=0.86) among older adults [40].

#### Plank

Participants were asked to hold a forearm plank on either their toes or knees, self-selected based on their ability, for as long as they were able to assess torso muscular endurance. Each participant was instructed to keep their elbows directly under their shoulders with forearms extended forward and a neutral spine and neck. The variation (ie, knees or toes) participants selected and total time participants were able to hold the plank were recorded. The plank assessment was not performed in cases where contraindications, such as cardiovascular concerns or upper extremity injuries, were present. Telehealth plank assessment has demonstrated good reliability (ICC=0.97) among adults [41].

#### Chair Sit and Reach

Hamstring flexibility was assessed using the chair sit and reach test. Participants sat on a chair near the front edge of the seat with one leg extended (ie, heel on the floor and foot dorsiflexed at approximately 90 degrees) and the other leg bent with the sole of the foot flat on the floor. Then they were asked to place one hand on top of the other with palms facing down. Participants were then instructed to slowly bend forward at the hips, keeping their back flat, as they reached down the extended leg as far as they could. A score was assigned based on how far participants were able to reach: a 2 for the toes, 1.5 for the ankle, 1.0 for the shin, 0 for anything above the shin. The chair sit and reach test has demonstrated good reliability (ICC=0.95) and validity among older adults [35,42].

#### Shoulder Range of Motion

Range of motion in the shoulder joint was assessed by measuring shoulder flexion, shoulder extension, and shoulder abduction. Range of motion for each movement was observed and visually estimated to the nearest 10 degrees during the telehealth initial assessment. Visual estimation of shoulder range of motion has demonstrated acceptable reliability (ICC=0.57‐0.70) among adults [43].

#### Clock Test

The clock test is a modified back scratch test used to assess shoulder internal rotation. Participants were instructed to reach behind their back with their palm facing out with the goal of reaching their hand as far up their back as possible. The test was conducted on both the right and left sides. The test is scored by visually estimating the position of the arm in correspondence to a position on the face of a clock during the telehealth initial assessment. Scores range from six to eleven on the right and six to one on the left with eleven and one indicating the highest levels of shoulder flexibility, respectively.

### Statistical Analysis

Descriptive statistics were reported as means and SDs or medians and IQRs for continuous variables and frequencies and percentages for categorical variables. Differences in age, BMI, and continuous initial assessment variables between older adults with cancer who did and did not complete a follow-up assessment were determined using independent samples *t* tests. Differences in categorical demographic, clinical, and initial assessment variables were assessed using chi-square tests. Among the older adults with cancer that completed a follow-up assessment, mean change variables were computed as the difference between the follow-up and baseline values. Missing assessment data were excluded case-wise to maximize the sample size for each variable. Change scores were compared to values considered to be the MCID. The 30-second chair stand test was the only assessment with a published MCID value [35]. Paired samples *t* tests were conducted to determine if there were significant differences in assessment outcomes following the exercise intervention. Cohen *d* effect sizes are reported as an indicator of effect size. A Cohen *d* of 0.2 was considered a small effect, 0.5 was considered medium, and 0.8 was considered large. For categorical outcomes mean change scores and 95% CIs were calculated to determine change across the intervention. All data were analyzed in SPSS (version 29.0; IBM Corp).

### Ethical Considerations

The protocol and waiver of informed consent was approved by the University of Utah Institutional Review Board (IRB_00072431) in accordance with the Declaration of Helsinki. All data presented were deidentified using study identification numbers prior to analysis. Compensation was not included for this study.

## Results

### Participants

A total of 68 older adults with cancer completed an initial assessment via telehealth and participated in the POWER program between March 2020 and December 2021. Older adults with cancer who participated in POWER via telehealth were 71.8 (SD 5.3) years of age on average (range 65‐92 y) and had a median BMI of 26.7 kg/m^2^ (IQR 7.3; Table 1). Most older adults with cancer were female (n=45, 66.2%) and were not actively receiving treatment during their participation in POWER (n=40, 58.8%). The most common cancer types among older adults were breast (n=18, 26.5%), prostate (n=13, 19.1%), and multiple myeloma (n=8, 11.8%).

**Table 1.** Participant demographic and clinical characteristics.

Variable			Total sample (N=68)	Follow-up assessment completed (n=20)	Follow-up assessment not completed (n=48)	Baseline differences between groups, *P* value
Age (years), mean (SD)			71.8 (5.3)	72.8 (4.6)	71.4 (5.5)	.33
BMI (kg/m^2^), median (IQR)			26.7 (7.3)	26.7 (7.3)	26.2 (7.1)	.62
**Sex, n (%)**						.90
	Male		23 (33.8)	7 (35)	16 (33.3)	
	Female		45 (66.2)	13 (65)	32 (66.7)	
**Race, n (%)**						—^a^
	White		68 (100)	20 (100)	48 (100)	
**Ethnicity, n (%)**						.25
	Non-Hispanic		65 (95.6)	20 (100)	45 (93.8)	
	Hispanic		3 (4.4)	0 (0)	3 (6.3)	
**Cancer stage, n (%)**						.72
	I		12 (17.6)	3 (15)	9 (18.8)	
	II		16 (23.5)	3 (15)	13 (27.1)	
	III		18 (26.5)	6 (30)	12 (25)	
	IV		10 (14.7)	3 (15)	7 (14.6)	
	Unstaged		11 (16.2)	5 (25)	6 (12.5)	
	Unknown		1 (1.5)	0 (0)	1 (2.1)	
**Active treatment^b^** **, n (%)**						.04^c^
	Yes		28 (41.2)	12 (60)	16 (33.3)	
	No		40 (58.8)	8 (40)	32 (66.7)	
**Treatment history^d^** **, n (%)**						
	**Chemotherapy**					.04^c^
		Yes	38 (55.9)	15 (75)	23 (47.9)	
		No	30 (44.1)	5 (25)	25 (52.1)	
	**Hormone therapy**					.78
		Yes	29 (42.6)	8 (40)	21 (43.8)	
		No	39 (57.4)	12 (60)	27 (56.3)	
	**Immunotherapy**					.67
		Yes	18 (26.5)	6 (30)	12 (25)	
		No	50 (73.5)	14 (70)	36 (75)	
	**Surgery**					.81
		Yes	49 (72.1)	14 (70)	35 (72.9)	
		No	19 (27.9)	6 (30)	13 (27.1)	
	**Radiation**					.20
		Yes	26 (38.2)	10 (50)	16 (33.3)	
		No	42 (61.8)	10 (50)	32 (66.7)	
**Number of treatment types, n (%)**						
	None		3 (4.4)	0 (0)	3 (6.2)	—
	Unimodal^e^		12 (17.7)	3 (15)	9 (18.8)	.71
	Bimodal^f^		19 (27.9)	6 (30)	13 (27.1)	.81
	Multimodal^g^		34 (50)	11 (55)	23 (47.9)	.60
**Cancer type, n (%)**						.62
	Bladder		2 (2.9)	0 (0)	2 (4.2)	
	Brain		1 (1.5)	1 (5)	0 (0)	
	Breast		18 (26.5)	5 (25)	13 (27.1)	
	Colon		2 (2.9)	0 (0)	2 (4.2)	
	Endometrial		3 (4.4)	1 (5)	2 (4.2)	
	Fallopian tube		2 (2.9)	0 (0)	2 (4.2)	
	Gallbladder		1 (1.5)	0 (0)	1 (2.1)	
	Kidney		2 (2.9)	0 (0)	2 (4.2)	
	Leukemia		1 (1.5)	1 (5)	0 (0)	
	Lung		1 (1.5)	0 (0)	1 (2.1)	
	Lymphoma		2 (2.9)	1 (5)	1 (2.1)	
	Melanoma		1 (1.5)	0 (0)	1 (2.1)	
	Multiple myeloma		8 (11.8)	3 (15)	5 (10.4)	
	Multiple cancer types		2 (2.9)	1 (5)	1 (2.1)	
	Ovarian		4 (5.9)	3 (15)	1 (2.1)	
	Peritoneal		2 (2.9)	0 (0)	2 (4.2)	
	Prostate		13 (19.1)	4 (20)	9 (18.8)	
	Rectal		1 (1.5)	0 (0)	1 (2.1)	
	Squamous cell carcinoma		1 (1.5)	0 (0)	1 (2.1)	
	Uterine		1 (1.5)	0 (0)	1 (2.1)	

a Not able to detect differences between groups.

b Active treatment: receiving any curative treatment during participation in the Personal Optimism With Exercise Recovery program.

c Statistical significance (*P*<.05).

d Treatment history: receiving the treatment type at any point in their care.

e Unimodal: 1 treatment type.

f Bimodal: 2 treatment types.

g Multimodal: 3 or more treatment types.

Of the 68 older adults who completed an initial assessment and participated in POWER, 29.4% (n=20) completed a telehealth follow-up assessment. The median time elapsed between initial and follow-up assessments was 16.5 weeks (IQR 5.75). The majority of older adults with cancer who completed a follow-up were on active treatment (n=12, 60%). Statistically significant differences were not observed among the following clinical and demographic variables among older adults with cancer who did and did not complete a follow-up assessment: age, BMI, sex, race, ethnicity, cancer stage, history of hormone therapy, immunotherapy, surgery, and radiation, number of treatment types, or cancer type. A statistically significant difference was observed for the proportion of older adults with cancer who reported being on active treatment (*P*=.04) and having received chemotherapy (*P*=.04) between those who did and did not complete a follow-up assessment.

### Change in Measured Outcomes

Values for each measured outcome from the initial telehealth assessment are reported in Table 2. There were no significant differences in initial assessment outcomes between the follow-up and no follow-up groups (*P*>.05).

**Table 2.** Initial assessment data.

Variable		Follow-up assessment completed		Follow-up assessment not completed		Between group difference	Total sample	
		n	Mean (SD)	n	Mean (SD)	*P* value	n	Mean (SD)
Standing march		19	77 (35.4)	42	77.2 (33.3)	.98	61	77.1 (4.3)
30-s maximum push-up		20	12.9 (4.3)	38	13.2 (3.5)	.77	58	13.1 (3.8)
30-s chair stand		19	12.3 (6.7)	42	12.7 (5.8)	.84	61	12.6 (6)
Plank (s)		14	79.1 (43.4)	31	63.8 (57.2)	.38	55	68.6 (53.3)
**Single leg stance (s)**								
	Right	19	29.2 (26.9)	33	20.8 (23.8)	.25	52	23.8 (25.1)
	Left	19	25.7 (26.5)	33	26.7 (24.5)	.89	52	26.4 (25)
**Shoulder flexion (degrees)**								
	Left	20	168 (8.8)	44	164.4 (16.5)	.37	64	165.6 (14.6)
	Right	20	165.8 (20)	44	165.9 (16)	.97	64	165.9 (17.2)
**Shoulder extension (degrees)**								
	Left	20	58.5 (9.6)	43	59.4 (12.2)	.78	63	59.1 (11.3)
	Right	20	58 (9.1)	43	59.9 (11)	.50	63	59.3 (10.4)
**Shoulder abduction (degrees)**								
	Left	20	171.3 (12.1)	44	168.5 (17.1)	.51	64	169.3 (15.6)
	Right	20	169.3 (21.5)	44	168.9 (18.2)	.94	64	169 (19.1)
**Clock test**								
	Left	20	3.5 (3)	39	4.5 (3.4)	.27	59	4.2 (3.3)
	Right	20	9.4 (1.1)	40	8.5 (2.6)	.52	60	8.8 (2.2)
**Seated sit and reach**								
	Left	20	1.4 (0.6)	42	1.6 (0.4)	.16	62	1.6 (0.5)
	Right	20	1.4 (0.6)	42	1.6 (0.5)	.40	62	1.5 (0.5)

Change in measured outcomes are reported in Table 3. Statistically significant changes were observed for the 30-second chair stand test (mean change +2.00 repetitions, 95% CI 0.12 to 3.88, Cohen *d*=0.51) and 30-second maximum push-up test (mean change +2.85 repetitions, 95% CI 1.60 to 4.11, Cohen *d*=1.06). Nine (47.3%) older adults with cancer had a change in 30-second chair stand scores that exceeded the MCID of 2.0 repetitions [35], and 14 (73.7%) older adults maintained their 30-second chair stand scores across the intervention. Although not statistically significant, positive changes were observed for the 2-minute standing march (mean change +12.79 repetitions, 95% CI −0.64 to 26.22, Cohen *d*=0.46), single leg stance on the left (mean change +4.80 s, 95% CI −0.67 to 10.27, Cohen *d*=0.44) and right (mean change +1.0 s, 95% CI −8.04 to 10.05, Cohen *d*=0.06), and shoulder abduction on the left (mean change +2.25 degrees, 95% CI −3.75 to 8.25, Cohen *d*=0.18) and right (mean change +0.25 degrees, 95% CI −4.34 to 4.84, Cohen *d*=0.03). The results from univariate analysis of covariance paralleled results from paired samples *t* tests.

**Table 3.** Change in assessment variables across the exercise intervention.

Variable		Quantity, n	Mean change	95% CI	*P* value	Cohen *d^a^*
Standing march		19	12.79	−0.64 to 26.22	.06	0.46
30-s maximum push-up		20	2.85	1.60 to 4.11	<.001^b^	1.06
30-s chair stand		19	2	0.12 to 3.88	.04^b^	0.51
Plank (s)		13	−5	−22.51 to 12.51	.55	0.17
**Single leg stance (s)**						
	Left	18	4.8	−0.67 to 10.27	.08	0.44
	Right	18	1	−8.04 to 10.05	.82	0.06
**Shoulder flexion (degrees)**						
	Left	20	−1.25	−6.56 to 4.06	.63	0.11
	Right	20	−3.5	−8.54 to 1.54	.16	0.33
**Shoulder extension (degrees)**						
	Left	20	−0.75	−4.64 to 3.14	.69	0.09
	Right	20	−0.5	−3.97 to 2.97	.76	0.07
**Shoulder abduction (degrees)**						
	Left	20	2.25	−3.75 to 8.25	.44	0.18
	Right	20	0.25	−4.34 to 4.84	.91	0.03
**Clock test**						
	Left	20	1.7	−0.96 to 4.33	—^c^	—
	Right	20	−0.53	−1.73 to 0.68	—	—
**Seated sit and reach**						
	Left	20	0.13	−0.11 to 0.36	—	—
	Right	20	0.15	−0.09 to 0.39	—	—

a Cohen *d* interpretation: small=0.2, medium=0.5, and large=0.8.

b Statistical significance (*P*<.05).

c Not applicable.

## Discussion

### Principal Findings

This study aimed to evaluate the preliminary effectiveness of a hospital-based telehealth exercise oncology program on physical function, muscular endurance, balance, and flexibility among older adults with cancer. Our findings demonstrate that supervised, one-on-one telehealth exercise may positively influence physical function among older adults with cancer. Additionally, nearly half (n=9) of individuals who completed a follow-up assessment exceeded an MCID change in the 30-second chair-stand test, a marker of lower extremity function.

### Comparison to Prior Work

The majority of research surrounding telehealth supervised exercise programs for older adults without [44,45] and with cancer [24,27] has focused on group exercise. Less is known about one-on-one telehealth exercise. Among older adults without cancer, participating in at least 12-weeks of supervised group telehealth exercise training prevents declines in physical function [44,45]. Among older adults with cancer, a feasibility study by Sattar et al [24] evaluated an 8-week group telehealth strength and balance training program and observed significant improvements in five time chair stand test scores. Additionally, Gell et al [27] carried out a pilot trial examining a 16-week group telehealth aerobic and resistance training program and observed significant improvements in 30-second chair stand test scores. Collectively, group telehealth exercise programming among older adults with and without cancer is effective at improving physical function.

Findings from this study contribute to the literature by addressing an important gap in our understanding regarding the effectiveness of one-on-one telehealth exercise among older cancer survivors. Among cancer survivors of all ages (range 14‐83 y), effectiveness of one-on-one supervised telehealth exercise has been evaluated [22]. Following 12-weeks of one-on-one training with a cancer exercise trainer once per week, cancer survivors significantly improved cardiovascular endurance, muscular endurance, and flexibility [22]. Findings from our study support previous research suggesting one-on-one telehealth exercise programs may positively influence physical function among older adults with cancer. Without exercise intervention, we would expect to see little to no change in physical function parameters over short durations in older adults living with cancer. Over 13-weeks Mikklesen et al [11] found a mean change of +0.4 repetitions in the 30-second chair stand test and −1.0 points in self-reported physical function among older cancer survivors receiving standard of care and no exercise intervention. Over longer durations (eg, ≥1 y) functional declines are greater and can persist for years following diagnosis [6]. Preventing declines in physical function is important in this population because physical function has been shown to have a protective effect against all-cause mortality in older adults with cancer [8].

In addition to examining the effect of one-on-one, supervised telehealth exercise on physical function in older adults with cancer, we also characterized older adults with cancer who chose to participate in a telehealth exercise program. This information adds to our body of knowledge by demonstrating that older adults with cancer can engage in one-on-one telehealth delivered exercise programs. Additionally, we observed that a significantly greater proportion of older adults with cancer who were on active treatment or had received chemotherapy completed a follow-up assessment. This finding suggests that older cancer survivors on active cancer treatment are willing to engage in telehealth exercise which is important as recent recommendations from the American Society of Clinical Oncology encourage cancer survivors on active treatment to participate in aerobic and resistance exercise [46]. Understanding who we are reaching with telehealth exercise programs, who may be missing, and who completes a telehealth follow-up assessment can inform the development of interventions to improve the engagement and reach of telehealth delivered exercise programming among cancer survivors of all ages.

### Strengths and Limitations

To our knowledge, this is one of the first studies that has evaluated effectiveness of one-on-one telehealth exercise programming exclusively among older adults with cancer and characterized the older adults who used this programming. We consider this a strength of our work. Additionally, the use of an established hospital-based exercise oncology program with over 15 years of experience offering telehealth exercise ensured high-quality exercise programming in this study. However, our study is not without limitations. First, the low follow-up assessment completion rate resulted in a small sample size and an underpowered analysis to demonstrate statistically significant changes in all outcomes measured. Therefore, the findings from this study are preliminary and additional research with a larger sample is needed. Second, the retrospective design of this study may have resulted in selection bias of participants who were more motivated to exercise and follow-up. Highly motivated individuals may be more likely to complete a follow-up assessment in the program potentially confounding the effects of the telehealth exercise program. Third, baseline and follow-up assessments were scheduled based on the participants’ availability which resulted in different staff conducting baseline and follow-up assessments for some participants. However, a small team of certified exercise physiologists administered all virtual assessments, adhering to standardized program procedures to minimize interrater variability. Fourth, the lack of a nonexercise control group limits the conclusions that can be made regarding the ability of telehealth exercise to prevent declines in physical function. Future work should consider a prospective study design and inclusion of potential confounding variables as covariates to determine the effectiveness of one-on-one telehealth exercise on markers of physical function.

### Conclusions

In summary, older adults living with and beyond cancer are able to participate in an exercise oncology program delivered via telehealth. Our findings provide preliminary evidence that telehealth may be a beneficial tool to facilitate exercise program delivery among older adults following a cancer diagnosis. However, telehealth exercise should not be considered a one-size-fits-all all approach as in-person, telehealth, or a combination of the two may be a better fit for some older adults with cancer, based on their needs and preferences. Further research is needed to understand the magnitude of the effects of one-on-one, supervised telehealth exercise on physical function among older adults with cancer.

## Abbreviations:

HCI: Huntsman Cancer Institute
ICC: intraclass correlation
MCID: minimal clinically important difference
POWER: Personal Optimism With Exercise Recovery
